# Title, Table of Contents and Acknowledgements

**DOI:** 10.1080/26410397.2020.1854503

**Published:** 2021-01-04


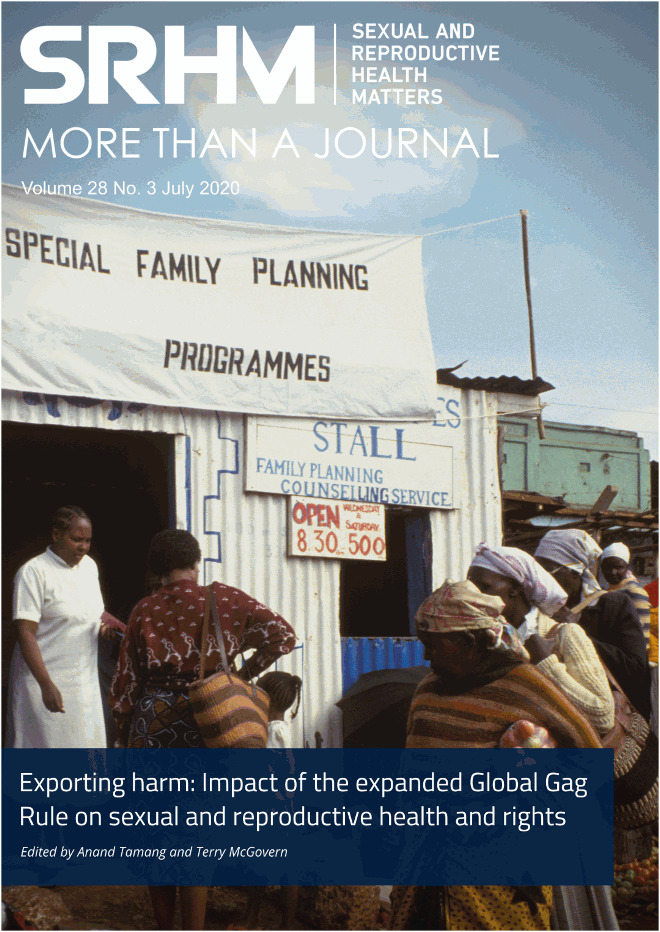


**Editorial**

1 *Terry McGovern, Anand Tamang* Exporting bad policy: an introduction to the special issue on the Global Gag Rule's impact

**Research articles**

5 *Jyotsna Tamang, Aagya Khanal, Anand Tamang, Naomi Gaspard, Maggie Magee, Marta Schaaf, Terry McGovern, Emily Maistrellis* Foreign ideology vs. national priority: impacts of the US Global Gag Rule on Nepal’s sexual and reproductive healthcare system

23 *Boniface Ayanbekongshie Ushie, Kenneth Juma, Grace Kimemia, Maggie Magee, Emily Maistrellis, Terry McGovern, Sara E Casey* Foreign assistance or attack? Impact of the expanded Global Gag Rule on sexual and reproductive health and rights in Kenya

39 *Lantonirina Ravaoarisoa, Mamy Jean Jacques Razafimahatratra, Mamy Andrianina Rakotondratsara, Naomi Gaspard, Marie Rolland Ratsimbazafy, Jean Florent Rafamantanantsoa, Voahanginirina Ramanantsoa, Marta Schaaf, Anne-Caroline Midy, Sara E Casey* Slowing progress: the US Global Gag Rule undermines access to contraception in Madagascar

54 *Terry McGovern, Marta Schaaf, Emily Battistini, Emily Maistrellis, Kathryn Gibb, Sara E Casey* From bad to worse: global governance of abortion and the Global Gag Rule

**Commentaries**

64 *Evelyne Opondo* Perspectives of an SRHR advocate on the impact of the Global Gag Rule in Kenya

68 *Meghan C Gallagher, Jamie M Vernaelde, Sara E Casey* Operational reality: the Global Gag Rule impacts sexual and reproductive health in humanitarian settings

71 *Beirne Roose-Snyder, Brian Honermann, Tambudzai Gonese-Manjonjo* Call in the lawyers: mitigating the Global Gag Rule

75 *Patty Skuster, Ram Chandra Khanal, Ernest Nyamato* Relics of imperialism: US foreign policy on abortion in the COVID Era

**Perspective**

79 *Shreejana Bajracharya* Adolescent and youth responses to the Global Gag Rule in Nepal

**Editor-in-Chief:** Julia Hussein

**Chief Executive:** Eszter Kismödi

**Managing Editor:** Pete Chapman

**Monitoring Editor:** Pathika Martin

**South Asia Hub Manager:** Sanjeeta Gawri

**Communications Manager:** Jessica MacKinnon

**Communications Officer:** Alexane Bremshey

**Operations Manager:** Edna Epelu / Amy Griffiths

**Associate Editors:** Laura Ferguson, Nambusi Kyegombe, Atsumi Hirose, Emma Pitchforth, Mindy Jane Roseman, Nina Sun, Joyce Wamoyi

Peer reviewers:

Sruthi Chandrasekaran, Bergen Cooper, Barbara Crane, Suchitra Dalvie, Kate Hesel, Candace Johnson, Risa Kaufman, Jedidah Maina, Janet Meyers, Roger Rochat, Lilian Sepulveda, Jennifer Sherwood, Patty Skuster, Jamie Vernaelde

Acknowledgements

Thank you to the Hewlett Foundation and the David and Lucile Packard Foundation.

Special thanks to Sara Casey for her thoughtful and consistent guidance.

Funding

This issue has been funded by a generous grant from the David and Lucile Packard Foundation to the Global Health Justice and Governance Program, Mailman School of Public Health, Columbia University.

**Cover photo:** Family planning clinic operating in a busy Kenyan market where women can visit while shopping. (c) Peter Barker, Panos Pictures.

Translation:

Françoise de Luca-Lacoste translated abstracts from English to French and Lisette Silva translated abstracts from English to Spanish.

Copyright © 2020

**Sexual and Reproductive Health Matters**. This is an Open Access journal distributed under the terms of the Creative Commons Attribution License (http://creativecommons.org/licenses/ by/4.0/), which allows for sharing and adapting the work for any purpose, even commercially, provided appropriate credit is given with a link to the originally published item, a reference to the author(s) and links to their homepages, reference to the license under which the article is published and a link to this, as well as an indication of any changes that have been made to the original.

ISSN (Online) 2641-0397

SRHM in translation online

Selected papers from the SRHM journal are published in Arabic, Chinese, French, Hindi, Portuguese, Russian and Spanish. Go to: http://www.srhm.org/our-journals/

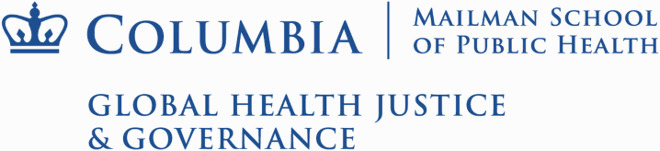

www.srhm.org / www.srhmjournal.org Twitter @SRHMJournalFacebook @SRHMJournal

